# Vitamin D inhibits lymphangiogenesis through VDR-dependent mechanisms

**DOI:** 10.1038/srep44403

**Published:** 2017-03-17

**Authors:** Saleh Yazdani, Fariba Poosti, Luis Toro, Johannes Wedel, Rik Mencke, Katarina Mirković, Martin H. de Borst, J. Steven Alexander, Gerjan Navis, Harry van Goor, Jacob van den Born, Jan-Luuk Hillebrands

**Affiliations:** 1 Department of Internal Medicine, Division of Nephrology, University of Groningen, University Medical Center Groningen, Groningen, The Netherlands; 2 Department of Pathology and Medical Biology, Division of Pathology, University of Groningen, University Medical Center Groningen, Groningen, The Netherlands; 3 Seccion de Nefrologia, Departamento de Medicina, Hospital Clinico Universidad de Chile, Santiago, Chile; 4 Centro de Investigacion Clinica Avanzada, Hospital Clinico Universidad de Chile, Santiago, Chile; 5 Department of Molecular and Cellular Physiology, Louisiana State University Health Sciences Center-Shreveport, Louisiana, USA

## Abstract

Excessive lymphangiogenesis is associated with cancer progression and renal disease. Attenuation of lymphangiogenesis might represent a novel strategy to target disease progression although clinically approved anti-lymphangiogenic drugs are not available yet. VitaminD(VitD)-deficiency is associated with increased cancer risk and chronic kidney disease. Presently, effects of VitD on lymphangiogenesis are unknown. Given the apparently protective effects of VitD and the deleterious associations of lymphangiogenesis with renal disease, we here tested the hypothesis that VitD has direct anti-lymphangiogenic effects *in vitro* and is able to attenuate lymphangiogenesis *in vivo. In vitro* cultured mouse lymphatic endothelial cells (LECs) expressed VitD Receptor (VDR), both on mRNA and protein levels. Active VitD (calcitriol) blocked LEC tube formation, reduced LEC proliferation, and induced LEC apoptosis. siRNA-mediated VDR knock-down reversed the inhibitory effect of calcitriol on LEC tube formation, demonstrating how such inhibition is VDR-dependent. *In vivo,* proteinuric rats were treated with vehicle or paricalcitol for 6 consecutive weeks. Compared with vehicle-treated proteinuric rats, paricalcitol showed markedly reduced renal lymphangiogenesis. In conclusion, our data show that VitD is anti-lymphangiogenic through VDR-dependent anti-proliferative and pro-apoptotic mechanisms. Our findings highlight an important novel function of VitD demonstrating how it may have therapeutic value in diseases accompanied by pathological lymphangiogenesis.

In addition to the blood vessels, lymphatic vessels (LVs) also form an integral part of the mammalian vasculature. LVs play crucial roles in a variety of vital processes in the body including immune surveillance, regulation of tissue fluid balance, lipid absorption and tissue homeostasis^1^. LVs are also associated with various pathological conditions such as inflammation, lymphedema, transplant rejection, hypertension, and tumor metastasis^2^. In the past decade, extensive research has described on the roles and mechanisms of LV function in organ homeostasis, both in health and disease^3^. Evidence from many animal models and clinico-pathological studies clearly support the importance of intra-organ LVs, and their outgrowth (i.e., lymphangiogenesis) in several solid organs including the kidney^4^. Renal lymphangiogenesis has been reported to occur in several conditions such as primary renal fibrotic diseases, proteinuria, and in renal allografts after transplantation^4^. Although not yet investigated in renal disease, modulating lymphangiogenesis in other organs by either promoting or inhibiting the formation of new LVs has been shown to have therapeutic value. Experimental lymphangiogenic therapy by vascular endothelial growth factor-C (VEGF-C) and angiopoietin-2 (ANG-2) restored lymphatic function, improved interstitial fluid drainage, and consequently reduced post-surgical lymphedema^5^. In contrast, inhibiting lymphangiogenesis in experimental transplantation models of cornea (by several strategies e.g. anti-vascular endothelial growth factor receptor 3 (VEGFR3) or anti-VEGF-C antibodies, neuropilin-2 blockade)^6^,^7^,^8^, heart (adenoviral VEGFR-3-Ig (*i.e.* soluble VEGFR3) and anti-VEGFR3 neutralizing antibody)^9^ and pancreatic islet (analog of sphingosine 1-phosphate FTY720, kinase inhibitor Sunitinib, anti-VEGFR3 antibody)^10^ increased graft survival. Targeting lymphangiogenesis has also been suggested to be a useful therapeutic approach in inflammatory^11^,^12^,^13^ and fibrotic diseases^14^,^15^, in addition to its well-studied central role in cancer therapy and tumor metastasis^16^,^17^. Despite numerous studies in this field, no anti-lymphangiogenic drugs have so far been approved for clinical use in these conditions.

Vitamin D (VitD) participates in many biological actions in the body, and hence is regarded as “*a pleiotropic hormone*”^18^. Beyond its cognate function in bone metabolism and calcium homeostasis, “*nonclassical*” effects of VitD have been shown protective in a variety of pathophysiological conditions such as cardiovascular disease, cancer and immunity^19^. These effects have been mostly linked to the expression of VitD Receptor (VDR) in tissues that are not classically involved in calcium homeostasis^19^. The ability of VitD to modulate the expression of genes involved in biological processes such as cell proliferation, apoptosis, oxidative stress, inflammation, matrix homeostasis and angiogenesis, makes VitD an attractive candidate therapeutic for cancer and cardiovascular diseases^20^,^21^. Blood vascular endothelial cells express VDR^22^ and VitD deficiency has been shown to be associated with increased arterial stiffness and endothelial dysfunction, underscoring the importance of VitD for maintaining endothelial health and function^23^.

Conversely, the data regarding the effect of VitD on angiogenesis is equivocal. Depending on the model, VitD analogues have been shown to have no effect on angiogenesis^24^,^25^, to have pro-angiogenic effects^26^, or to have anti-angiogenic effects^27^. However, no data have been reported on the potential effects of VitD on lymphatic endothelial cells (LECs) and lymphangiogenesis. The impact of VitD and its analogues on renal pathophysiology has been extensively studied^28^,^29^,^30^,^31^,^32^,^33^. Most of the results from these studies suggest that VitD may be considered a generally safe and effective therapeutic in diverse kidney diseases, but the effect of VitD on renal lymphatics and lymphangiogenesis has not yet been described.

Given the beneficial effects of VitD treatment in renal disease, and the deleterious associations of lymphangiogenesis with renal disease, we here tested the hypothesis that VitD has direct anti-lymphangiogenic effects and is able to attenuate lymphangiogenesis.

To address this, we here examined the direct effects of VitD on LEC tube formation, proliferation and apoptosis *in vitro*, and the effects of VitD treatment on lymphangiogenesis *in vivo*.

## Results

### Cultured lymphatic endothelial cells (LECs) express VDR

**Figure 1:**
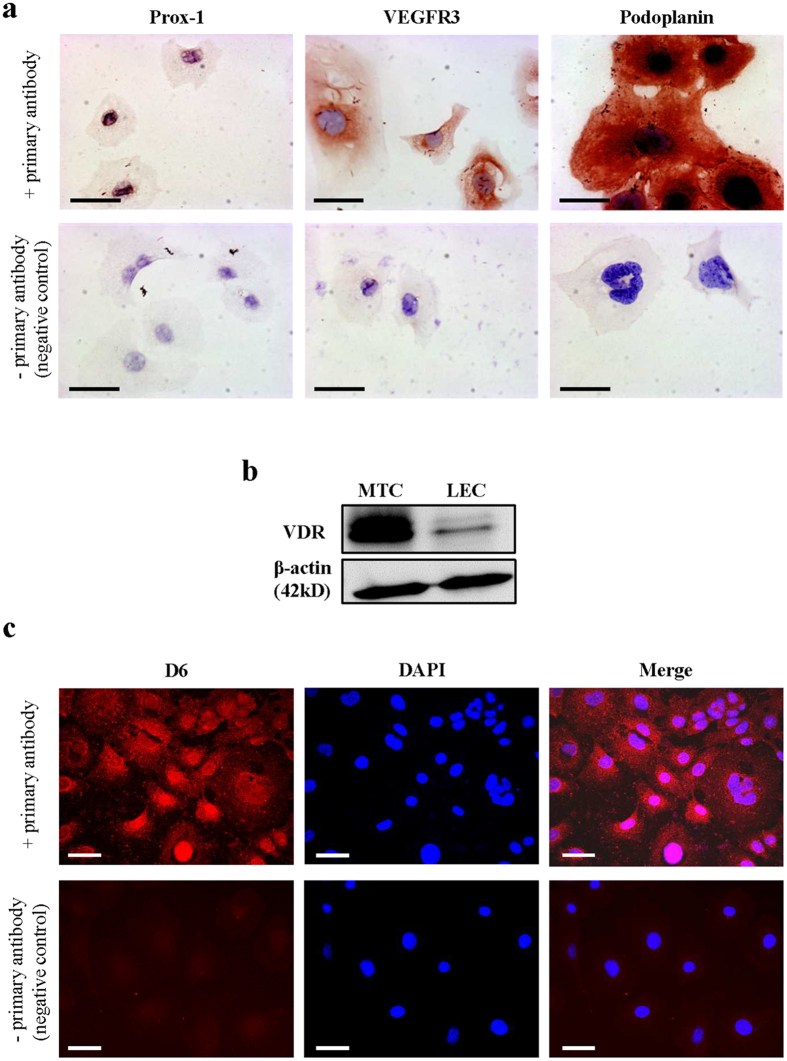
Cultured murine LECs express VDR. To check lymphatic origin of mouse LECs, we used three different well-established markers expressed by LECs. (**a**) Lymphatic origin of murine LECs was confirmed by IHC for Prox-1, VEGFR3 and Podoplanin (400x). Scale bar: 50 μm. (**b**) VDR expression of *in vitro* grown murine LECs was evaluated by western blot. Murine renal tubular epithelial cells (MTCs) served as positive control. (**c**) VDR expression of murine LECs was assessed by immunofluorescence (antibody D6) staining (200x). Scale bar: 50 μm.

Immunohistochemistry (IHC) was performed to confirm lymphatic endothelial phenotype of cultured cells. As shown in Fig. 1a, compared to the negative control staining, LECs expressed Prospero homeobox protein 1 (Prox-1), VEGFR3, and Podoplanin. This combination of markers is expressed exclusively by LECs. The presence of VDR protein was confirmed by western blotting using anti-VDR antibody D6 (Fig. 1b). Mouse tubular epithelial cells (MTCs) were considered positive controls since mouse renal tubular epithelium is known to express VDR^34^. Western blot results indicate that LECs clearly express VDR albeit at lower levels compared with MTCs. Immunofluorescent staining with the same antibody confirmed VDR expression in LECs (Fig. 1c).

### Calcitriol inhibits tube formation of LECs in a VDR-dependent manner

**Figure 2:**
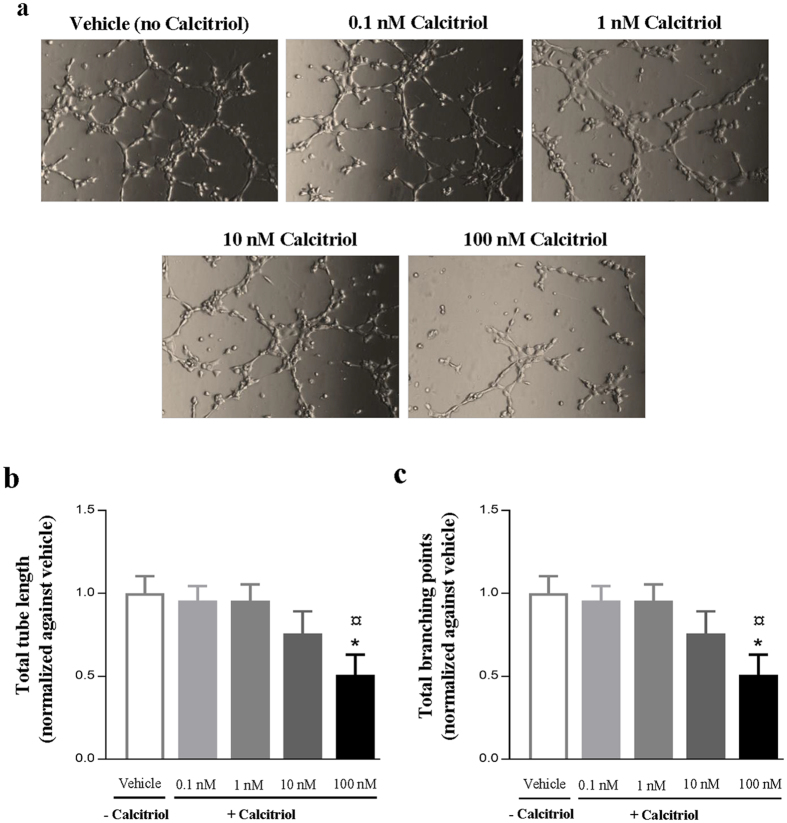
Calcitriol blocks LEC tube formation *in vitro*. LECs were seeded into angiogenesis slides incubated with indicated calcitriol concentrations for 4 hours. (**a**) Representative images (10x magnification) of tube formation by LECs treated with different calcitriol concentrations. (**b**) The total tube length and (**c**) number of branching points of 5 high power fields per condition was quantified and normalized against the vehicle control. The data are expressed as the mean ± SEM from three independent experiments. *P < 0.01 compared to the control (vehicle) group, ^¤^P < 0.01 compared to 0.1 nM.

**Figure 3:**
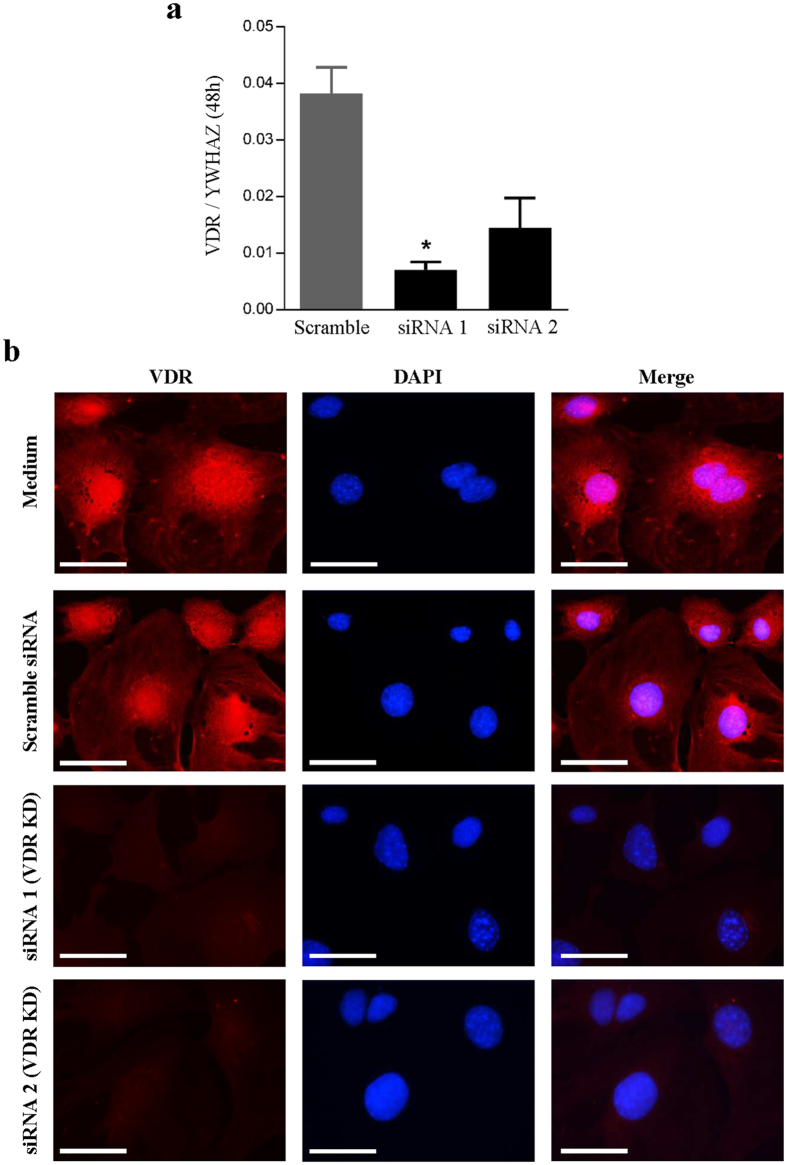
siRNA for VDR sufficiently knocks down VDR expression in cultured LECs. LECs were transfected with siRNA for VDR (siRNA 1 and siRNA 2) and scrambled siRNA. (**a**) mRNA expression of VDR measured at 48 h after transfection. siRNA 1 and 2 largely abolished VDR mRNA expression compared with scrambled siRNA transfection. (**b**) Photomicrographs of immunofluorescence for VDR (mouse anti-VDR D6) 48 h after siRNA transfection. The staining showed that VDR expression was not affected in scramble-transfected cells (compared with medium), whereas VDR expression was virtually absent in siRNA VDR-transfected LECs (400x). Scale bar: 50 μm.

**Figure 4:**
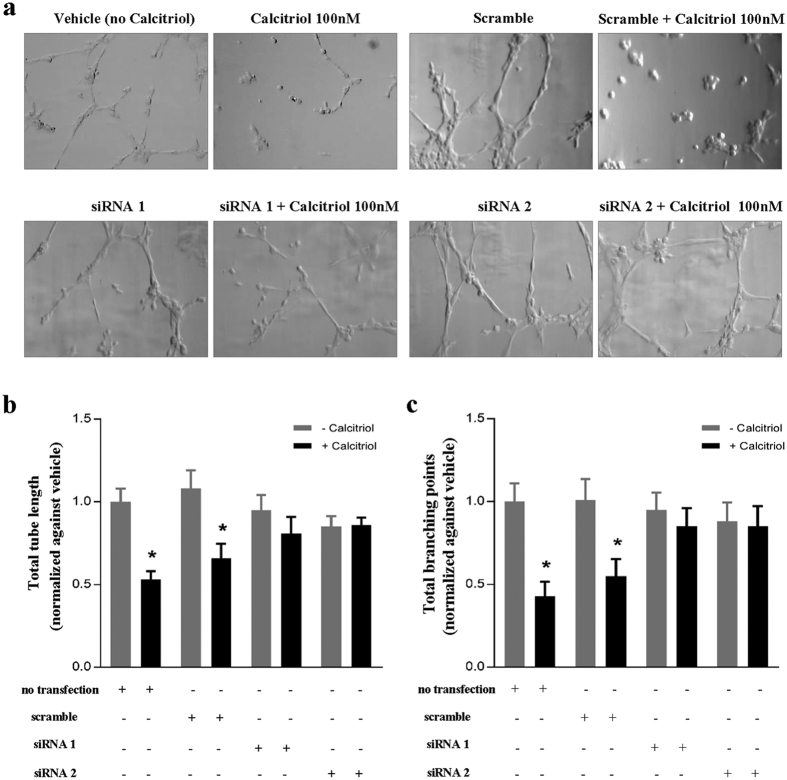
The inhibitory effect of calcitriol on tube formation is VDR-dependent. LECs without siRNA-transfection (no transfection), LECs with scramble siRNA-transfection, and with VDR siRNA-transfection (siRNA 1 and siRNA 2) were seeded into angiogenesis and treated with 100 nM calcitriol or PBS for 4 h. (**a**) Representative images (10x magnification) of tube formation. (**b**) Total length of tube-like structures and (**c**) number of branching points quantified in 5 high power fields and normalized against non-transfected, vehicle (no calcitriol)-treated LECs. Data are expressed as the mean ± SEM from three independent experiments. *P < 0.05 when compared to the non-transfected and vehicle treated control group.

To investigate a potential lymphangiostatic effect of calcitriol on LECs, tube formation assays were performed. Interestingly, calcitriol appeared to have lymphangiostatic effect, and dose-dependently decreased lymphatic capillary tube formation (Fig. 2a). Quantitative analyses expressed as both total tube length (Fig. 2b) and total number of branching points (Fig. 2c) revealed reduced tube formation in the presence of calcitriol. These data indicate that the VitD analogue calcitriol exerts anti-lymphangiogenic effects *in vitro*. We next examined whether the observed effects on tube formation were VDR-dependent. At mRNA level, qRT-PCR indicated that VDR expression in LECs transfected with VDR siRNA was reduced remarkably compared to scramble siRNA knock-down after 48 h (81% and 62% reduction compared to scramble in siRNA 1 and siRNA 2, respectively) (Fig. 3a). Immunofluorescent staining showed that VDR siRNA transfected cells had virtually no VDR protein expression when compared with non-transfected and scramble siRNA transfected cells (Fig. 3b). Using VDR and scramble siRNA transfected cells in the tube formation assay, we showed that VDR knock-down almost completely abolished the inhibitory effect of calcitriol on tube formation (Fig. 4a). Quantification showed that the significant reduction of tube formation induced by calcitriol was abolished by VDR siRNA, both in terms of tube length (Fig. 4b) but also numbers of branching points (Fig. 4c). These data indicate that calcitriol-induced impairment of tube formation is VDR-dependent.

### Effects of calcitriol on LEC proliferation and apoptosis

**Figure 5:**
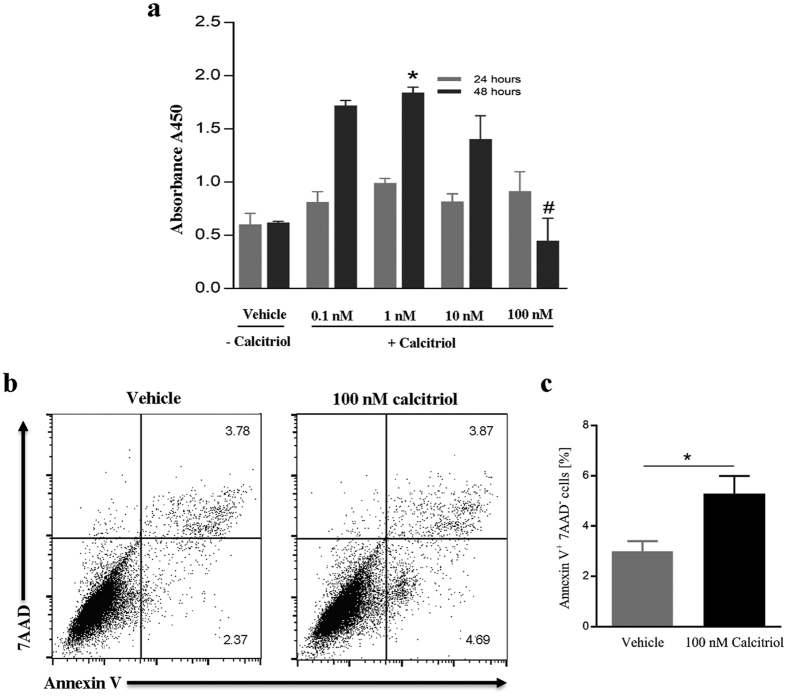
Calcitriol (100 nM) inhibits LEC proliferation and induces apoptosis. (**a**) LECs were plated in 96-well gelatin-coated plates (2,000 cell per well) and treated with PBS or indicated concentrations of calcitriol for 24 h or 48 h. Cell proliferation was quantified using the cell proliferation reagent WST-1 during the last 3 h of cell culture. Bars represent mean absorbance at 450 nm ± SEM of three independent experiments. Note that low doses (0.1–10 nM) of calcitriol promoted, while high doses (100 nM) inhibited proliferation. *P < 0.05 compared to the vehicle treatment, ^#^P < 0.05 compared with 1 nM calcitriol. (**b**) Apoptosis was analyzed by flow cytometry. LECs were treated with 100 nM calcitriol for 6 h and subsequently stained with anti-Annexin V and 7AAD. Apoptotic cells are Annexin V^+^ 7AAD^−^. Representative dot plots are depicted. (**c**) Quantitative analysis of five independent experiments. The data are expressed as the mean frequency of Annexin V^+^ 7AAD^−^ cells ± SEM. *P < 0.05.

Next we sought to identify the mechanism underlying VitD/VDR-mediated reduction in tube formation, focusing on LEC proliferation and induction of apoptosis. We studied the effect of calcitriol on LEC proliferation *in vitro* using Cell Proliferation Reagent WST-1. No effect of calcitriol on proliferation was observed after 24 h incubation (Fig. 5a). However, after 48 h, the lower calcitriol concentrations (0.1 nM, 1 nM, and 10 nM) showed a marked increase in proliferation compared with control. However, the highest concentration (100 nM), which had a clear inhibitory effect on tube formation, also significantly reduced this increase of proliferation to the level of placebo control.

We next analyzed possible induction of apoptosis. To accomplish this, LECs were plated and after 24 h of attachment and subsequent 24 h serum starvation, cells were treated with calcitriol (100 nM) or vehicle alone for 6 h. Untreated cells served as negative control (medium). To detect apoptotic cells staining for Annexin V and 7-AAD was performed followed by flow cytometry. Apoptotic cells are Annexin V^+^ 7-AAD^−^. Figure 5b shows representative dot plots of non-treated and calcitriol-treated LECs. Quantitative analysis indicated that 100 nM calcitriol induces significant LEC apoptosis (Fig. 5c).

### Paricalcitol treatment prevents lymphangiogenesis *in vivo*

**Figure 6:**
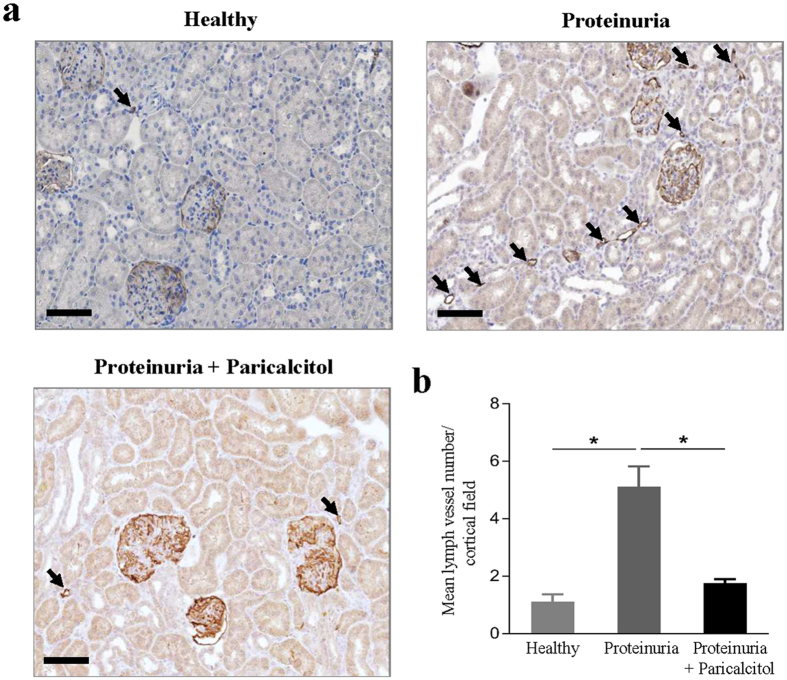
Paricalcitol prevents renal lymphangiogenesis in proteinuric rats. (**a**) At week 12, compared to healthy non-proteinuric control rats, the kidneys of proteinuric rats showed a marked increase in the number of lymphatic vessels. Representative sections are depicted; arrows point to LVs (all 200x). Scale bar: 100 μm. (**b**) Paricalcitol treatment significantly prevented renal lymphangiogenesis. Quantification of the lymphatic vessels number in cortical area of healthy non-proteinuric, vehicle-treated and paricalcitol-treated proteinuric rat kidneys. Bars represent mean LV number/cortical field ± SEM. *P < 0.05.

To translate our *in vitro* findings to the *in vivo* setting, we analyzed whether the VitD analogue paricalcitol is able to attenuate proteinuria-associated lymphangiogenesis in a rat model. We previously showed that in the Adriamycin Nephrosis rat model, massive lymphangiogenesis occurs between weeks 6 to 12 after induction of disease^35^. Therefore, we treated animals in this time frame with paricalcitol to test whether this can impair adriamycin-induced lymphangiogenesis in the kidney. The results of LV quantification in the kidney of proteinuric rats receiving paricalcitol (160 ng/kg three times per week p.o.) showed a significant inhibitory effect on renal lymphangiogenesis compared to vehicle-treated proteinuric rats (Fig. 6a,b). This experiment confirms, as a proof of principle, that treatment with a VitD analogue is also able to prevent lymphangiogenesis *in vivo*.

## Discussion

The present study reveals an inhibitory effect of vitamin D (VitD) on lymphangiogenesis. The results demonstrate that in the presence of active VitD (calcitriol), LEC tube formation and proliferation were attenuated whilst apoptosis was induced *in vitro*. VDR knock-down in LECs reversed the inhibitory effect of calcitriol on LEC tube formation, confirming that this effect is VDR-dependent. *In vivo*, paricalcitol treatment significantly decreased lymphangiogenesis in the kidneys of adriamycin-induced proteinuric rats. This inhibitory effect of VitD may have important clinical implications in disease conditions which exhibit excessive or inappropriate as well as detrimental lymphangiogenesis.

Numerous studies have highlighted the fundamental role of lymphatic vessels and lymphangiogenesis in physiological and pathological conditions^1^,^2^,^3^. Both blocking and promoting lymphangiogenesis have been suggested to be advantageous, and therefore imply to be highly context-dependent. For example, inducing lymphangiogenesis has been shown to decrease interstitial fluid accumulation in experimental models of lymphedema^36^,^37^, and also reported to be of advantage in the resolution of inflammation^11^,^12^,^38^. A recent study by Chi *et al*.^39^ showed that promoting lymphangiogenesis by VEGF-C156S increased clearance of interstitial hyaluronan (HA), and could improve graft outcomes in an animal model of lung transplantation. Contrary to these findings, inhibiting lymphangiogenesis in several experimental transplantation models improved graft survival^6^,^7^,^8^,^9^,^10^. Whether lymphangiogenesis is a ‘friend or foe’ in kidney transplantation, is still unclear^4^. Lymphangiogenesis has also sparked a great deal of interest in cancer therapy. Numerous studies have demonstrated the feasibility of anti-lymphangiogenic therapy to prevent tumor metastasis^1^,^2^,^3^,^16^,^40^. Nevertheless, despite the long history of research on the mechanism of lymphangiogenesis and encouraging experimental results, clinically approved anti-lymphangiogenic drugs are still not available.

VitD has become a focus of intense interest both because of its vital roles in health, but also as an important immune modulator. The beneficial therapeutic effects of VitD and VitD analogues have been proposed in many conditions, ranging from proteinuria, fibrosis, atherosclerosis and inflammation to cancer therapy^28^,^29^,^30^,^31^,^32^,^33^,^41^. It has been found that VDR regulates at least 229 genes through binding to 2776 genomic DNA binding sites. These genes target anti-proliferative, pro-apoptotic, anti-inflammatory, angiostatic, immune regulatory and pro-differentiation functions, and affect many cell functions in tissue- and cell-specific manners^41^.

Here we demonstrated the expression of VDR in mouse LECs *in vitro*, both at the mRNA and protein expression levels. Consistent with these findings, the inhibitory effect of LEC tube formation was completely blocked after gene knock-down of VDR in LECs, highlighting the dependence of functional VDR in mouse LECs for reduced lymphangiogenesis. Taken together, these experimental findings shown that calcitriol blocks LEC tube formation in a VDR-dependent manner. Blood endothelial cells also express VDR^22^, and active VitD has been shown to induce both angiogenic and anti-angiogenic effects on blood endothelial cells^24^,^25^,^26^,^27^,^42^,^43^,^44^,^45^. The effect of VitD on blood endothelial cell proliferation is also contradictory. Calcitriol has been shown to inhibit the proliferation of human umbilical vein endothelial cells (HUVEC)^46^, while another report found an induction of proliferation through a nitric oxide-dependent mechanism in the same cell type^26^. These inconsistencies might reflect differences in treatment concentrations or origin of cells/tissues that were used.

The effect of calcitriol on LEC proliferation and apoptosis was studied in order to gain additional insights into mechanisms underlying the VitD-mediated suppression of LEC tube formation. Our results showed a concentration-dependent effect of calcitriol on LEC proliferation. While the lower concentrations promoted proliferation, the highest used concentration significantly prevented this increase of proliferation. Such a biphasic effect of the VitD on proliferation has also been reported in mouse epidermal keratinocytes^47^,^48^ where low concentrations of VitD promoted proliferation while higher concentration (10–1000 nM) inhibited proliferation. This result may provide important information regarding physiological and pharmacological actions of VitD, respectively. The same calcitriol concentration (100 nM) also significantly blocked LEC tube formation, which may explain, at least in part, the underlying mechanism(s). One might argue that the high (100 nM) VitD might have toxic effects and therefore impairs tube formation and proliferation, and promotes apoptosis. This is however unlikely since VDR-knock down fully restored tube formation even in the presence of 100 nM calcitriol.

Programmed cell-death or apoptosis, a key mechanism in cancer therapy, can be also induced by VitD through repressing the expression of anti-apoptotic proteins such as Bcl2 and Bcl-X, as well as by inducing the expression of pro-apoptotic proteins such as BAX, BAD, and BAK^49^,^50^,^51^,^52^. Although in current study we did not delve deeply into detailed mechanisms of apoptosis, we did find that calcitriol significantly induced apoptosis in LECs, which might be another reason for capillary tube suppression in the presence of calcitriol.

Moreover, treatment with the VitD analogue paricalcitol in the *in vivo* proteinuric adriamycin-induced nephrosis model significantly blocked development of renal lymphangiogenesis compared to vehicle-treated rats. Paricalcitol is a synthetic VitD_2_ agonist of the VDR, and is considered a selective VDR activator^53^,^54^. Although the affinity of paricalcitol for the VDR is reported to be about three times less than that of calcitriol, calcemic and phosphatemic effects of paricalcitol are 10 times lower^55^. Consequently, we preferred to use paricalcitol which has more selective VDR activity and lower risk of side effects such as increase in calcium concentrations or hypercalcemia (in very high concentrations). Our *in vivo* observations are again consistent with our data on the inhibitory effect of calcitriol *in vitro*.

In summary, in this work we show for the first time the expression of VDR by lymphatic endothelial cells. Our observations provide further information that calcitriol-induced inhibition of LEC tube formation may reflect the anti-proliferative and pro-apoptotic effects of VitD in this model. More in depth and mechanistic studies are still required to elucidate the molecular mechanisms responsible for the observed inhibitory effects of VitD on LECs. In keeping with our *in vitro* observations, we also showed the anti-lymphangiogenic effect of paricalcitol in the adriamycin-induced nephrosis rat model. These findings describe potentially important novel therapeutic effects of VitD since blocking lymphangiogenesis could be useful in treating conditions exhibiting detrimental, unfavorable lymphangiogenesis.

## Materials and Methods

### Cell lines and cultures

Murine lymphatic endothelial cells (LECs) were originally derived from mesenteric adventitial tissue isolated from transgenic mice expressing a temperature-sensitive SV40 large T antigen (H-2Kb-tsA58 mice)^35^. LECs were grown in gelatin-coated flasks using high glucose Dulbecco’s Modified Eagle’s Medium (DMEM, Cat. No. BE12-709E, Lonza, Belgium) supplemented with 10% FBS, 4 mM glutamine (Cat. No. 25030, Invitrogen-Gibco, Bleiswijk, The Netherlands), and penicillin/streptomycin (Penicillin 50 U/ml/Streptomycin 50 μg/ml, Cat. No. 15070-063, Invitrogen-Gibco, Bleiswijk, The Netherlands) at 37 °C and 5% CO_2_. Mouse tubular epithelial cells (MTCs)^36^ were grown in high glucose DMEM supplemented with 10% FBS, 25 mM Hepes (Cat. No. 15630, Invitrogen-Gibco), 50 μg/mL penicillin/streptomycin, and 2 mM glutamine at 37 °C and 5% CO_2_.

### mRNA isolation and qRT-PCR analysis

Total RNA was extracted from MTCs and LECs using RNeasy Micro kit (Qiagen, The Netherlands). RNA concentrations were measured with a Nano Drop spectrophotometer (NanoDrop Technologies, Wilminton, DE, USA). For MTCs, cDNA was synthesized using QuantiTect Reverse Transcriptase Kit (Qiagen, The Netherlands) according to the manufacturer’s guidelines. For LECs cDNA synthesis, 1 μg RNA was converted to cDNA using SuperScript II reverse transcriptase and random hexamers (Life Technologies) according to the manufacturer’s instructions. PCR reactions were executed in a 10 μl reaction volume containing 1× Taqman Gene Expression Assay mix (Applied Biosystems, Foster City, USA) and 1× qPCR master mix (Eurogentec, Liege, Belgium). The Taqman primers were as follows: YWHAZ Mm03950126_s1 (Applied Biosystems, Carlsbad, USA) and VDR Mm00437297_m1 (Applied Biosystems, Carlsbad, USA). For qRT-PCR reactions, an ABI7900HT thermal cycler (Applied Biosystems, Foster City, CA, USA) was used. YWHAZ (tyrosine 3-monooxygenase/tryptophan 5-monooxygenase activation protein ζ) was used as housekeeping gene, and relative gene expression was calculated using the 2^−ΔΔCt^ method.

### Western blotting

To determine VDR expression in MTCs and LECs at the protein level, western blotting was performed. Cells were washed with the Tris-buffered saline (TBS, 20 mM Tris, pH 7.5, 150 mM NaCl) and homogenized in Radio immunoprecipitation assay buffer (50 mM Tris–HCl, 150 mM NaCl, 0.1% SDS, 0.1% Igepal in 0.5% sodium deoxycholate with 1 tablet of protease inhibitor cocktail and 1 tablet of phosphatase inhibitor; Roche Diagnostics, Mannheim, Germany). The lysates were mixed with loading buffer to make a total volume of 20 μl. Samples were then spun down, boiled at 100 °C for 5 min in a thermo block, spun down again and put on ice. 20 μl of every sample was loaded on a denaturizing 7.5% polyacrylamide gel. On every gel, a protein ladder was included to indicate band size. The gel was run for 15 min at 40 V and 75 min at 120 V. After running, the gel was soaked for 10 minutes in blotting buffer. The gel was then transferred on a nitrocellulose membrane followed by blotting at 150 mA for 45 min. Afterwards the membrane was blocked with TBS with 5% milk powder and 0.1% Tween 20 for 2 h at 4 °C. The membrane was incubated overnight at 4 °C with mouse anti-VDR D6 (1:500) (Santa Cruz Biotechnology, Inc) diluted in TBS-T (TBS with 0.1% Tween 20) with 1% milk powder. The membrane was washed twice followed by incubation for 2 h at RT with rabbit anti-mouse Ig horseradish peroxidase (HRP) (DAKO, Heverlee, Belgium) diluted 1:1000 in TBS-T with 1% milk powder. The membrane was washed twice and HRP activity was visualized with a chemiluminescence kit (NEL103E001EA, PerkinElmer^®^) according to the manufacturer’s instructions. The chemiluminescence membrane was recorded on an ImageQuant™ LAS 4000 system.

### Immunohistochemistry (IHC) and immunofluorescence (IF)

Immunostaining was performed on formalin-fixed, paraffin-embedded tissues or acetone-fixed cultured cells/tissues. Three-micron paraffin-sections were deparaffinized followed by heat-induced epitope retrieval (HIER) in boiling 10 mM Tris–1 mM EDTA buffer (pH 9.0) for 10 min. After cooling, tissue endogenous peroxidase was blocked using 0.3% H_2_O_2_ in phosphate-buffered saline (PBS). For frozen tissue sections, endogenous peroxidase was blocked using 0.03% H_2_O_2_ in PBS. Endogenous biotin was inhibited using biotin blocking system (Code X0590, DAKO, Heverlee, Belgium). For staining on *in vitro* cultured LECs, cells were seeded at a density of 5 × 10^3^ cells/ml on glass coverslips (Marienfeld laboratory glassware, Lauda-Königshofen, Germany) in 12-well plates overnight. For IHC and IF, cells or tissue cryosections were acetone-fixed (10 min. RT) followed by permeabilization in 0.1% Triton-X100 in PBS at RT. Cells (no endogenous blockade was applied) or tissue sections were then incubated with the following primary antibodies for 1 h at RT: hamster anti-mouse podoplanin (Cat. No. DM3501, Acris Antibodies Inc., Germany), mouse-anti human podoplanin, clone D2–40 (Code M3619, DAKO, Heverlee, Belgium), rabbit-anti human prox-1 (Cat. No. 102-PA32S, ReliaTech GmbH, Germany), mouse anti-rat podoplanin (Cat. No. 11–035, Angio Bio, Del Mar, USA), goat anti-mouse VEGFR3 (Cat. No. AF743, R&D system, Inc.), mouse anti-VDR (D-6: sc-13133, Santa Cruz Biotechnology, Inc.). Following washing in 0.1% Tween 20 in PBS (PBS-T), cells or tissue sections were incubated with appropriate secondary antibodies diluted in PBS + 1% mouse or human serum for 30 min. The following secondary antibodies (all purchased from DAKO, Heverlee, Belgium) were used: biotinylated rabbit anti-mouse Ig, rabbit anti-mouse Ig horseradish peroxidase (HRP)-conjugated, rabbit anti-mouse Ig fluorescein isothiocyanate (FITC)-conjugated, rabbit anti-goat Ig HRP-conjugated, goat anti-rabbit Ig and goat-anti mouse Ig (both HRP and FITC-conjugated). The TSATM Tetramethylrhodamine System (10 min, PerkinElmer LAS Inc., USA) and FITC-conjugated streptavidin (45 min, Invitrogen) were used to visualize HRP-conjugated and biotinylated secondary antibodies, respectively. As a control for non-specific secondary antibody binding, PBS + 1% BSA substituted primary antibodies (negative control). 4′,6-diamidino-2-phenylindole (DAPI) (Vector Laboratories, Inc.) was used for nuclear staining. IF was visualized on a Leica fluorescent microscope (DM4000B, equipped with DFC345FX camera and LAS software) or Zeiss confocal microscope (LSM 780 Axio Observer.Z1, Carl Zeiss Microscopy GmbH, Jena, Germany).

IHC was performed on paraffin sections using the same protocol as described above but HRP-conjugated secondary antibodies were diluted in PBS + 1% normal rat serum. HRP-activity was visualized using 3,3′-diaminobenzidine (DAB, Sigma-Aldrich, USA) as chromogen with hematoxylin counterstaining. Slides were scanned on a Nano Zoomer HT whole slide scanner (Hamamatsu Photonics K.K., Shizuoka Pref., Japan) and sections were analyzed using Aperio Image Scope software (Aperio Technologies Inc, Vista, CA, USA).

### Tube formation assay

Matrigel tube formation assays were performed to investigate lymphangiogenesis *in vitro*. The endothelial cell tube formation assay on basement membrane (including Matrigel) is widely used to study angiogenic potential *in vitro* (reviewed in ref. 56). Although vascular endothelial cells are most commonly used in this assay, it has also been described useful to study lymphangiogenesis^57^. Although not visible at the microscopical level, studies performed at the ultrastructural level (*i.e.* electron microscopy) revealed that the tubes formed when using either vascular endothelial cells^58^ or LECs^59^ contain a lumen. Based on this data the LEC tube formation assay appears to be a suitable model to study lymphangiogenesis *in vitro*. Here 15-well μ-Slide Angiogenesis (Cat. No. 81501, ibidi, Martinsried, Germany) and Growth Factor Reduced Matrigel (Cat. No. 356230, BD Biosciences, Bedford, MA) were used. 10 μL of Matrigel was placed per well and incubated at 37 °C for 30 minutes. 50 μL of 1.4 × 10^5^ cells/mL LECs incubated in DMEM were added to each well with or without 1α,25-Dihydroxyvitamin D_3_ (calcitriol) (D1530, Sigma Aldrich, USA). Calcitriol was added to the medium to reach a final concentration of 0.1 nM, 1 nM, 10 nM or 100 nM (performed in triplicates). Cells were incubated for 4 h at 37 °C in humidified air with 5% CO_2_. Tube formation assay was also performed on VDR knock-down cells. No siRNA-transfected, VDR siRNA-transfected and scramble siRNA-transfected LECs were seeded into μ-Slide Angiogenesis pre-coated with 10 μl growth factor-reduced Matrigel. Simultaneously, cells were treated with 100 nM calcitriol or PBS. After 4 h incubation the capillary tubes were analyzed. Representative images were captured with an Olympus inverted phase-contrast microscope (Olympus Optical C., Melviller, NY, USA) equipped with the Quick Imaging System. From each well 5 images were taken (at 100x magnification) at fixed areas (starting at the center of the well and then above, below, right, left of the center). To perform quantifications, both the total cumulative tube length (*i.e.* total cumulative length of all tubes visible in the photomicrographs) and the total number of branching points were determined in all 5 pictures per well and corrected by the image surface using Image J 1.46r software (Rasband, W.S., U.S. National Institutes of Health). Then the average of the 3 wells values per group was calculated. Results were normalized to the data of the vehicle-treated group (PBS). Three independent experiments were performed.

### siRNA transfection

To determine the efficacy of siRNA knock-down on VDR mRNA and protein expression levels in LECs, knock-down cells were analyzed at 48 h. Commercially available VDR siRNA (s75929 and s75930, Life Technologies, Bleiswijk, The Netherlands) and Silencer^®^ Select scramble siRNA (Cat. No. 4390843, Life Technologies) were used. As additional controls, untreated cells (medium only) and cells treated with only Lipofectamine^®^ Transfection Reagent (Cat. No. 11668-019, Life Technologies) without siRNAs were used. 5 nmole siRNA was diluted in RNase-free water to a final concentration of 2.5 μM. Transfections were performed in 12-well plates by adding 200 μl of Opti-MEM reduced serum medium and 4.8 μl of 2.5 μM siRNA/well. Next, 2 μl/well of lipofectamine was added and incubated for 10–20 min at RT. 1 ml of cells/medium was added per well (10 nM siRNA concentration) and incubated for 48 h at 37 °C, 5% CO_2_. All transfections were performed on LECs that had been cultured in antibiotics-free complete LEC medium. All experiments were performed 48 hours after transfection.

### Proliferation assay

The effect of 1α,25-Dihydroxyvitamin D_3_ (calcitriol) (D1530, Sigma Aldrich, USA) on proliferation of LECs was determined by plating cells in 96-well gelatin-coated plates (2,000 cells per well) in 200 μl medium. The cells were incubated at 37 °C for 3.5 hours for adherence and then treated with vehicle (PBS) or various concentrations of calcitriol for 24 h or 48 h. For cells cultured for 48 h (with or without calcitriol), medium was replenished with new medium containing the appropriate calcitriol concentration after 24 h. Cell proliferation was quantified using Cell Proliferation Reagent WST-1 (100 μl/well) according to the manufacturer’s instructions and absorption was measured after three hours of WST-1 incubation.

### Apoptosis assay

LECs were plated in 6-well culture plates in DMEM at a density of 1.5 × 10^5^ cells/ml. Cells were allowed to attach for 24 h, starved for additional 24 h and subsequently stimulated with 100 nM calcitriol for 6 h. Non-stimulated cells (no calcitriol, medium only) served as negative control. A positive control for the staining procedure was obtained by addition of 0.4 mM H_2_O_2_ to LECs for 6 h to induce cell death. Cells were trypsinized, washed in PBS twice and stained with anti-Annexin V Apoptosis Detection Kit and 7-AAD (Cat: 640922, Uithoorn, The Netherlands) according the supplier’s protocol. Fluorescence was assessed on a FACS Calibur flow cytometer (BD Bioscience, Breda, The Netherlands) within the same day. At least 10,000 gated events were collected per sample and data was analyzed using Flowjo software (Tree Star, Inc., Ashland, OR, USA).

### Induction of proteinuria in rats

Adriamycin Nephrosis (AN) was induced in Wistar rats (Harlan, The Netherlands) by a single injection of 1.5 mg/kg adriamycin (Doxorubicin^®^) into the tail vein as previously described^37^. After 6 weeks animals were placed in 24-hour metabolic cages for urine collection, and then rats were stratified according to the level of proteinuria into two experimental groups. Subsequently, at six weeks after adriamycin injection, renal biopsy was taken via abdominal incision in order to evaluate kidney histology and numbers of LVs. Animals were treated then with paricalcitol 160 ng/kg three times per week p.o. (paricalcitol/ethanol dissolved in water, n = 8) or vehicle (ethanol diluted in water, n = 6) from week 6 to 12. At the end of the study, blood and kidneys were collected for further analysis. All animals received care in compliance with the recommendations of ARRIVE, the Directive 2010/63/EU and the Dutch Law on Experimental Animal Care, and were approved by the animal ethics committee of the University of Groningen.

### Quantification lymphatic vessel density

In order to evaluate lymphangiogenesis in rat kidneys, podoplanin-stained kidney sections were scanned on a NanoZoomer HT machine (Hamamatsu Photonics K.K., Shizuoka Pref., Japan). After scanning, the number of podoplanin-positive vessels present in 30 cortical tubulointerstitial fields per kidney was manually counted using Aperio ImageScope software (version 9.1.772.1570, Aperio Technologies Inc, Vista, CA, USA) and Image J 1.46r software (Rasband, W.S., U.S. National Institutes of Health). The results are expressed as mean number of lymphatic vessels per cortical tubulointerstitial field ± SEM.

### Statistics

Statistical analysis was performed using GraphPad Prism 5.0 (GraphPad Software Inc, La Jolla, CA). Data are expressed as mean ± standard error of the mean (SEM). Differences between multiple groups were calculated using ANOVA (Bonferroni post-hoc test) with p < 0.05 as the minimal level of significance. Comparisons of two groups were performed using Mann-Whitney U-test.

## Additional Information

**How to cite this article**: Yazdani, S. *et al*. Vitamin D inhibits lymphangiogenesis through VDR-dependent mechanisms. *Sci. Rep.* **7**, 44403; doi: 10.1038/srep44403 (2017).

**Publisher's note:** Springer Nature remains neutral with regard to jurisdictional claims in published maps and institutional affiliations.
